# Mal-Positioning of Dialysis Catheter in Anomalous Left Superior Pulmonary Vein in a Patient with Acute Type A Dissection, a Case Report

**DOI:** 10.21470/1678-9741-2018-0254

**Published:** 2019

**Authors:** Tariq Minhas, Jane Bhaskara Pillai, Ting Lau

**Affiliations:** 1 Cardiothoracic surgery, University Hospital Coventry Ringgold Standard Institution - Coventry, United Kingdom of Great Britain and Northern Ireland

**Keywords:** Aortic Dissection, Partial Anomalous Pulmonary Vein Drainage, Renal Dialysis Catheter

## Abstract

The partial anomalous pulmonary vein drainage is a rare congenital defect. The pulmonary vein drains in to a systemic vein instead of draining in to the left atrium.

In this rare birth defect, the right sided pulmonary vein involvement is more prevalent than the left sided pulmonary veins.

We present a case where the anomalous left superior pulmonary vein was diagnosed when a renal dialysis catheter (size = 12F x 16cm) was mal-positioned in to the Anomalous left superior pulmonary vein, demonstrating confusing blood results. We describe how a systematic multidisciplinary approach and use of advanced imaging techniques can recognise and deal with this rare clinical dilemma.

**Table:** 

Abbreviations, acronyms & symbols	
ASD	= Atrial septal defect
CT	= Computed tomography
DO_2_	= Systemic oxygen delivery
FiO_2_	= Fraction of inspired oxygen
PAPVC	= Partial anomalous pulmonary venous connection
PAPVR	= Partial anomalous pulmonary venous return
PLSVC	= Persistent left sided superior vena cava
TAPVC	= Total anomalous pulmonary venous connection

## INTRODUCTION

Partial anomalous pulmonary venous return (PAPVR) is a rare congenital anomaly. The left superior anomalous pulmonary vein may connect the innominate vein directly through left vertical vein or may drain in to the coronary sinus, carrying the oxygenated blood to the venous circulation. Partial anomalous pulmonary venous return from the right lung is twice as common from the left sided partial anomalous pulmonary return.

In this case, the anomalous left superior pulmonary was diagnosed after an accidental mal-positioning of dialysis catheter into the anomalous left superior pulmonary vein.

The following congenital vascular anomalies should be considered in differential diagnosis.

### Partial Anomalous Pulmonary Venous Drainage

The pulmonary veins are four blood vessels which drain oxygenated blood from the lungs to the left atrium of the heart. Embryologically, the pulmonary veins arise separately and are absorbed into the left atrium to form its smooth internal surface. Total or partial anomalous pulmonary venous connection (TAPVC or PAPVC) is a rare heart defect in which all (total) or one or more (partial) pulmonary veins do not connect normally to the left atrium. The veins are re-directed to the right atrium or systemic veins by an abnormal connection. Total anomalous pulmonary circulation invariably exits with Atrial septal defect^[1]^ and causes varying degrees of symptoms including cyanosis. It needs an urgent correction surgery.

### Partial Anomalous Pulmonary Vein Connection

Partial anomalous pulmonary venous connection (PAPVC) is a rare congenital anomaly and has a reported prevalence of 0.4 - 0.7% and more frequently affects the right lung (80%) than the left lung^[2]^. The most common form of the partial anomalous pulmonary vein drainage is an anomalous right upper lobe vein that enters the Superior vena cava or right atrium^[3]^. While Partial anomalous pulmonary venous connection (PAPVC) may occur as an isolated anomaly, it is commonly associated with other congenital cardiac abnormalities. The most common congenital anomaly associated with Partial anomalous pulmonary venous connection (PAPVC) is an atrial septal defect (ASD), most commonly a sinus venosus type ASD^[4]^.

For the left upper lobe Partial anomalous pulmonary venous connection (PAPVC), it is commonly connected to the left brachiocephalic vein via a persistent left vertical vein. Oxygenated venous blood then returns to the right side of the heart. Depending on the amount of left to right shunt of blood flow through the anomalous venous connection, patients may present with symptoms of pulmonary hypertension, be mildly symptomatic or even asymptomatic. Asymptomatic patients are usually incidentally diagnosed much later in life.

### Persistent Left Sided Superior Vena Cava

Persistent left sided superior vena cava (PLSVC)^[4]^, which has got a prevalence of 0.3 - 0.5%, is the persistence of the left anterior cardinal vein which joins left horn of the sinus venosus, which later is recognised as coronary sinus. In the commoner variant, where the persistent left sided superior vena cava (PLSVC) drains into the RA only via the coronary sinus, the congenital which condition does not pose any symptomatic issues. In 10% of cases, the persistent left sided superior vena cava (PLSVC), on its way to the coronary sinus may be unroofed in the Left atrium, which also can show an arterial blood sample within a venous structure.

## CASE REPORT

A 58 year-old male patient presented with history of acute chest pain which was radiating to the back. He was investigated for acute myocardial infarction but on subsequent imaging he was found to have acute type A aortic dissection. The urgent surgery was planned. He underwent acute Stanford Type A aortic dissection repair.

On postoperative day 2, the patient was still intubated and ventilated. The urine output had dropped below 20mls an hour and the creatinine level were increased to 280 micromol/L. The blood gas PH:7.28, PO2: 8.3kpa, PCO2: 5.1, HCO_3_: 22 mEq/L and Lactate 3.2 mmol.

**Fig. 1:**
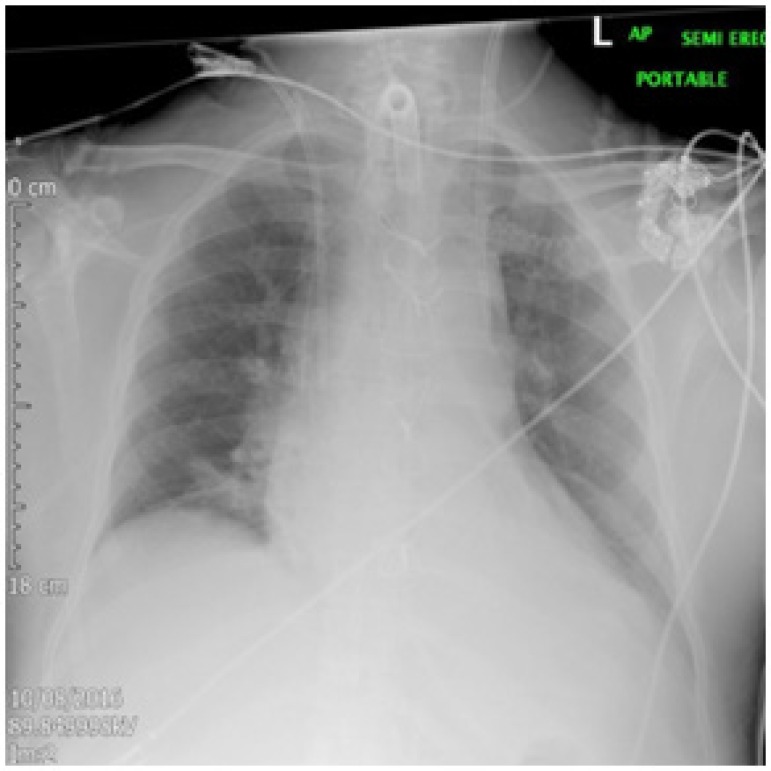
The chest x-ray shows mal-positioned dialysis catheter.

A conventional double lumen dialysis catheter insertion for continuous veno-venous haemodialysis through left internal jugular vein was performed by a consultant intensivist under Ultrasound guidance (Figure 1).

As clear from the check chest x-ray film, that the tip of the catheter is abnormally directed toward the hilum of the left lung, which normally should cross the midline to the opposite direction. After recognising the abnormal position of the catheter, the decision was made not to use the dialysis catheter until the confirmation of location and position is made.

The chest x-ray landmarks are not typical for the position of the left innominate vein. The tip of the dialysis catheter is at T6 level and is too close to the carina at the level of thoracic vertebrae 4/5. The left innominate vein should be much higher at thoracic vertebrae T2/3 level and it should only join the right innominate vein past the midline at thoracic vertebra T4 level. Therefore, the appearances cannot be accepted without further confirmation of the position of the catheter.

However, the patient remained stable since the line was inserted; no difficulties were experienced during the line insertion. The chest x-ray does not show a Pneumothorax, pleural or mediastinal collection.

An easy and fluent aspiration of the blood from the catheter confirmed the intravascular position, but the concern still persists.

Firstly, the blood from both dialysis catheter lumens was tested on a blood gas machine. The proximal lumen blood sample showed a venous, while the distal lumen blood sample showed an arterial gas result.

The suspicion of the proximal lumen into the vein and the distal lumen in an artery was a significant concern.

The further evaluation by using the standard pressure transducer was made. Both the lumens were separately connected to the pressure transducer and both showed the venous waveform trace. How do we reconcile these confusing findings? Before making any decision to remove the catheter, an opinion from the colleague radiologist was requested.

**Fig. 2:**
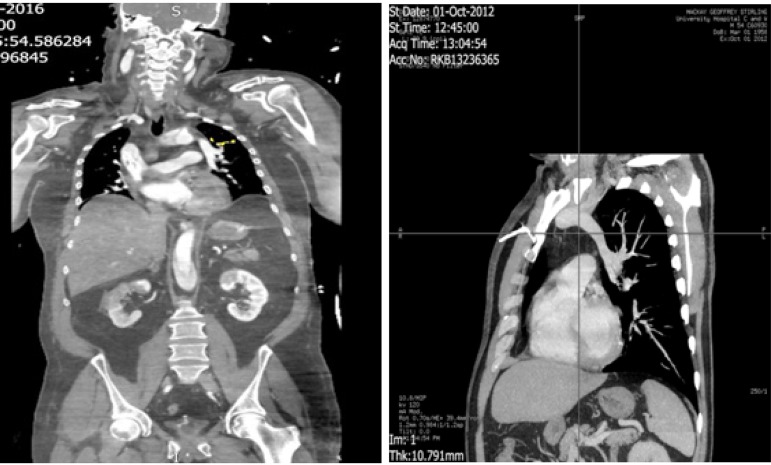
The contrast CT scan showing anomalous left superior pulmonary vein.

The contrast chest computed tomography (CT) (Figure 2) clearly shows the anomalous left superior pulmonary vein, which has been missed on preoperative CT scan.

**Fig. 3:**
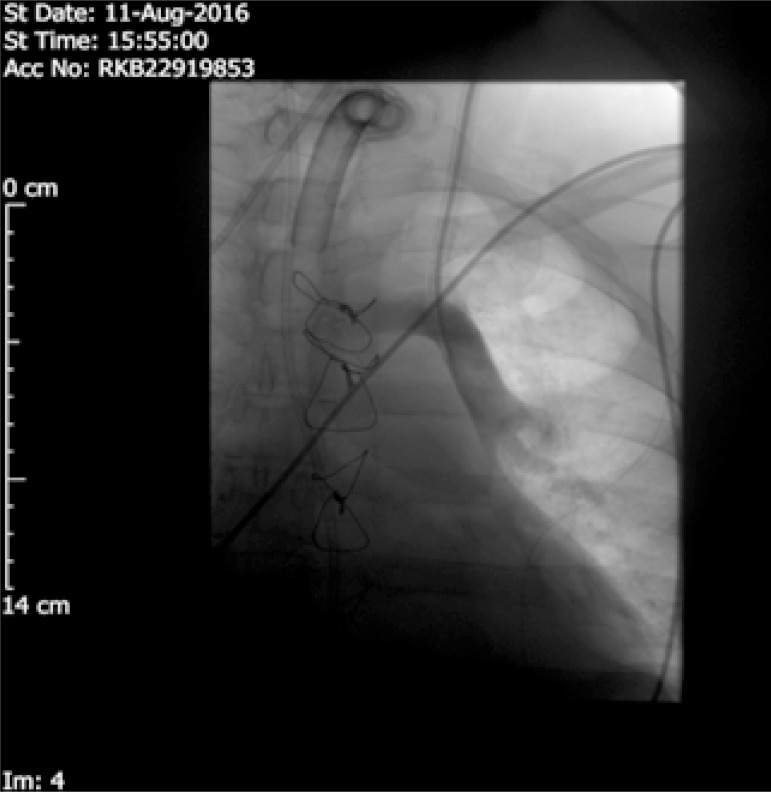
The Linogram shows the anomalous vein draining in left innominate vein.

On the radiologist's advice, a contrast Linogram (Figure 3) through the dialysis line was performed. Contrast was injected individually through both the lumens. The first image with the contrast injection down the proximal lumen showed the contrast moving horizontally and then to the right.

This confirms the catheter's position within the innominate vein. However, the second injection showed a contrast-filled structure that descends further down to the left para-mediastinal silhouette, but there was no extravasation of the contrast.

The two possible diagnoses that came to mind for a venous structure down the left para-mediastinal silhouette were a persistent Left sided Superior vena cava^[4]^ or an Anomalous left sided pulmonary venous drainage to the central systemic veins^[5]^. The Left sided superior vena cava should travel to the coronary sinus, that does not appear to be the case. Since the contrast was flowing towards the hilum of the left lung, followed by a branching pattern into the lung parenchyma confirmed the diagnosis of a partial anomalous left superior pulmonary vein. Moreover, the arterial blood gas via the distal dialysis central line supports the diagnosis. The sample of the blood collected from the proximal lumen confirms that the proximal lumen is in the left innominate vein.

A review of the preoperative CT scan was also done. Apart of the obvious acute Type A aortic dissection, the diagnosis of a coexisting pathology had been missed. Since, it was missed on preoperative scans it is extremely rare to encounter a silent congenital malformation. The Left superior pulmonary vein emerging from the lung hilum was clearly seen to ascend and join the left innominate vein. The anatomy of the remaining pulmonary venous drainage was normal.

## DISCUSSION

In this case, after the diagnosis was made, the left dialysis line was safely removed. The left side was not used again for central lines. There were no immediate complications related to this unusual situation.

Had the diagnosis been missed and the dialysis line used, the possibility of left upper lobe lung injury exists due to forced injection of fluid/blood into the pulmonary venous system with the possibility of pulmonary oedema or even haemorrhage.

In the absence of such congenital venous anomalies, with a similar radiological finding would have raised the concerns of left innominate vein injury, could have presented as left sided haemothorax, cardiovascular compromise and possibly a fatal outcome. The attention to the surgical technique at line insertion, the use of an ultrasound, the colour of the blood aspirated, the force of the flow of the blood via the central line are the immediate safeguards. To further assist or in case of doubt, the pressure transducer and the blood gas analysis should be used. Finally, every central line must be confirmed with chest x-ray. If any doubt exists, do not use the line and consult for further advice.

Incidentally, to our knowledge, there is no etiologic relationship between partial anomalous pulmonary circulation and acute aortic dissection. But co-existence has been described in the literature. Turner's syndrome is one example.

Retrospectively, it did explain intra-operative and post-operative haemodynamic instability. While weaning the patient off bypass after completion of the procedure, there was an elevation of the central venous pressure and a sluggish right heart response. However, in this case the added insult of the Left to Right shunt and pulmonary hypertension can further compound the problem. Although the lung fields were clear on the postoperative chest X-ray, there was an initial postoperative desaturation to 80-85% on 100% of fraction of inspired oxygen (FiO_2_). Initially, this was difficult to clearly explain. The patient did not have a coexisting ASD. We postulate the following hypothesis.

In light of the new finding of PAPVC, the effective systemic oxygen delivery (DO_2_) is reduced by as much as 20%. We did not calculate the shunt fraction. Effectively, in addition to the right lung, only half the left lung contributed to systemic oxygenation. Oxygen saturation can be explained as the ratio between the oxygen content to the maximum oxygen capacity of unit blood. Given the theoretical oxygen debt following deep hypothermic circulatory arrest and subsequent rise in VO_2_ post operatively, this mismatch could explain the desaturation, by the greater fall in oxygen content from increased extraction to the overall reduced systemic capacity.

The case also did emphasise to be comprehensive when reviewing clinical investigations such as the CT Scan beyond the most obvious.

## CONCLUSION

The presence of an anomalous isolated left superior vein drainage is a rare congenital defect. Team work and the use of new imaging technology can help in accurate diagnosis of this defect.

**Table:** 

Authors' roles & responsibilities	
TM	Substantial contributions to the conception or design of the work; or the acquisition, analysis, or interpretation of data for the work; final approval of the version to be published
JBP	Substantial contributions to the conception or design of the work; or the acquisition, analysis, or interpretation of data for the work; final approval of the version to be published
TL	Substantial contributions to the conception or design of the work; or the acquisition, analysis, or interpretation of data for the work; final approval of the version to be published
